# Parental perceptions and experiences of care in the surgical neonatal intensive care unit

**DOI:** 10.1007/s00383-023-05484-0

**Published:** 2023-06-01

**Authors:** Jennifer Y. Lam, Alexandra Howlett, Lori M. Stephen, Mary E. Brindle

**Affiliations:** 1 Division of Pediatric Surgery, Western University, Children’s Hospital–London Health Sciences Centre, B1-188, 800 Commissioners Rd. E, London, ON N6A 5W9 Canada; 2 Section of Neonatology, University of Calgary, Alberta Children’s Hospital, 28 Oki Dr. NW, Calgary, AB T3B 6A8 Canada; 3 Section of Pediatric Surgery, University of Calgary, Alberta Children’s Hospital, 28 Oki Dr. NW, Calgary, AB T3B 6A8 Canada

**Keywords:** Parental experience, Surgery, Neonatal, Parental perception

## Abstract

**Background:**

Parents endure significant stress when their newborns require surgery while in the neonatal intensive care unit (NICU). Our study aims to explore the surgical NICU experience from the parents’ perspective and identify areas that may improve this experience. A secondary objective was to integrate their feedback to refine the implementation strategy of the neonatal enhanced recovery after surgery (ERAS^®^) guideline.

**Methods:**

In December 2019, five surgical NICU parents participated in a focus group. Conversation surrounded parents’ perspectives and experiences of the surgical NICU. Inductive analysis was performed to identify data, themes, and concepts that emerged from the discussion.

**Results:**

Participants identified four major interrelated themes that impacted the surgical parents’ NICU experience. These themes include (1) parental state, both physical and emotional, (2) the altered parental caregiver role which necessitates identifying alternative meaningful parental experiences, (3) the care team dynamic, incorporating consistency and effective communication, and (4) the discharge process which may be significantly eased through graduated, hands-on training.

**Conclusion:**

Key elements of the neonatal ERAS^®^ guideline address major themes and stressors identified by parents. The parental perspective may help clinicians appreciate the parent surgical NICU experience and assist in improving family-centered care to surgical infants and their families.

## Introduction

Although the birth of a child is often a positive experience, new parents also experience stress, exhaustion, anxiety, and financial concerns [1–5]. Parental stresses are further magnified when infants have health concerns requiring care in the neonatal intensive care unit (NICU) [1–11]. An estimated 13.6% of all Canadian births in 2003–2004 required NICU admission [12]. While the majority of NICU admissions are related to prematurity, a significant proportion of neonates present with congenital anomalies or acquired conditions requiring surgery [3, 7, 8, 11].

The needs and stressors of parents of NICU infants have been studied, but primarily in the preterm population [1–11, 13]. Although there are many experiences shared by parents of premature infants and parents of infants undergoing surgery, parents of surgical infants are subject to a unique set of stressors related to complex surgical care, anesthetic risks, and surgical complications [1, 2, 4, 7, 8, 11]. Few studies explore the surgical parent’s experience, many of which are restricted to cardiac surgery, combine children of varying ages, and/or are remote from the NICU environment [1, 2, 7, 8]. Existing literature describing the parental experiences of non-cardiac surgical infants has reported survey data [1, 4, 7, 8, 11]. Qualitative study designs, such as those involving focus groups, allow for a deeper exploration of the personal experiences of parents.

Understanding parental needs and experiences allows for the development of meaningful family-centered care strategies in the NICU. These strategies are key to improving the outcomes of hospitalized neonates by raising awareness of care pathways, and ensuring clear communication, thus reducing harmful practice variation [14, 15]. The newly published neonatal enhanced recovery after surgery (ERAS®) guideline incorporates parental involvement in a holistic, multidisciplinary tool to optimize the recovery of surgical neonates and the experience of their families [16–18].

We hypothesize that by understanding the needs and stressors of surgical parents in the NICU, we can tailor our supports and communication strategies, and address deficiencies in our units thus providing improved holistic care to our surgical NICU patients and their families. Furthermore, a secondary objective of this study was to incorporate parent feedback about their specific needs in the surgical NICU to strengthen the implementation strategy of the neonatal ERAS® guideline at our institution.

## Methods

**Fig. 1:**
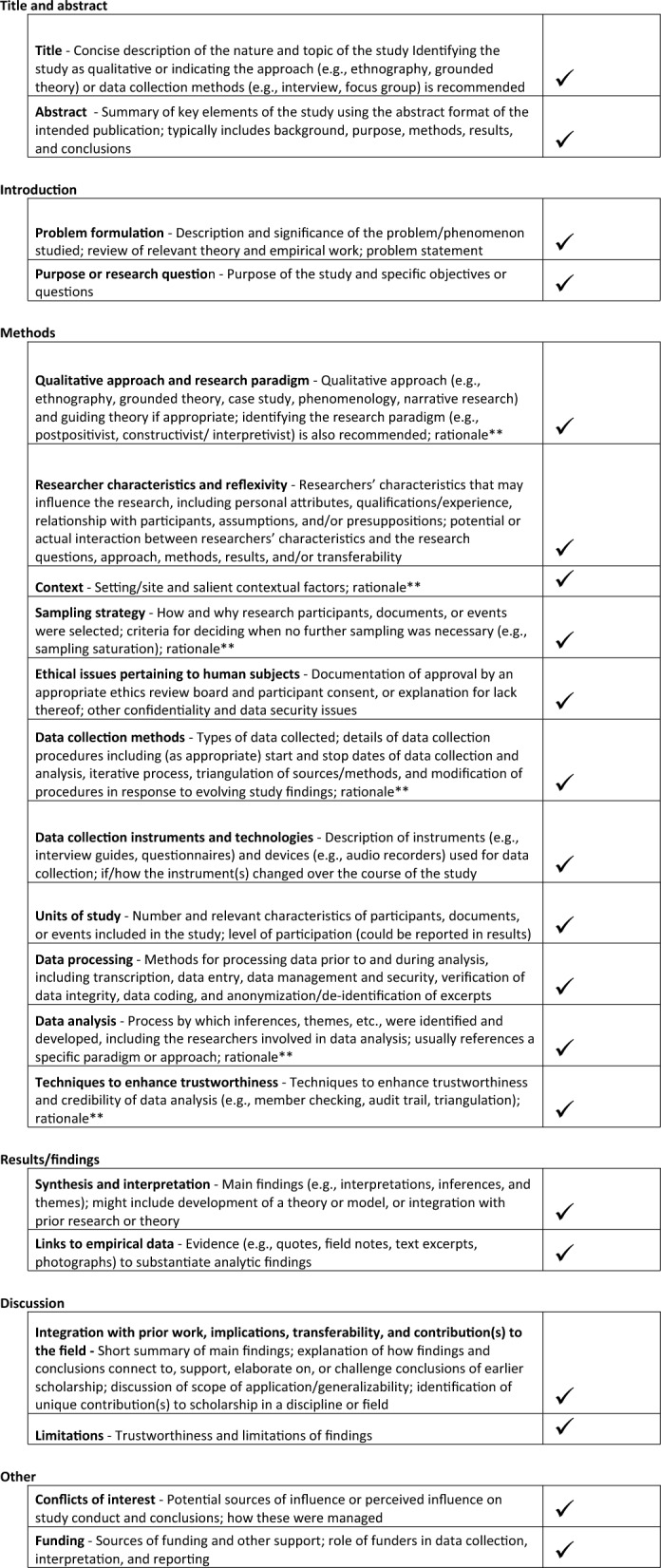
Standards for Reporting Qualitative Research Checklist [19]

This study was designed and followed the principles outlined by the Standards for Reporting Qualitative Research guidelines (Fig. 1) [19]. This study was initially created in relation to the novel neonatal ERAS^®^ guideline to engage parent advisors to inform the neonatal ERAS^®^ implementation strategy [16, 20].

After University of Calgary research board ethics approval (REB 18–0579), consenting parents of neonates who required a major general surgery procedure between August 7 and December 9, 2019 were recruited. As the outcomes of focus groups are contingent on participant discussion, parental English language proficiency was required to ensure meaningful focus group participation.

The study consisted of two phases. In December 2019, two authors (MB, JL) conducted an in-person focus group using a semi-structured script (Table 1). The script encouraged dialogue surrounding parental experiences and perspectives specific to their child’s NICU stay. Further areas of inquiry were parental involvement and suggestions for improving the parent–clinician partnership in relation to the neonatal ERAS^®^ guideline.

**Table 1:** Semi-structured focus group questions

1	How were you educated about your role in caring for your child after surgery? What information do you want to know? What did you find the most helpful? Is there anything you feel would have made it easier for you? Would you have wanted to be more or less involved?
2	What was your relationship with the care team?
3	What was communication like with the care team? What strategies do you think could improve communication between providers and parents?
4	Did you feel prepared at discharge? What were you most nervous about? What could help make this transition easier? What would make you feel more comfortable with being discharged?
5	How could we best prepare parents for their child’s discharge from the hospital? What educational strategies/tools would you find the most helpful?
6	Do you have any thoughts on the ERAS care program as a whole?
7	Part of the ERAS program revolves around engaging parents to be participants in their child’s care through early involvement and purposeful practicing of skills. Are there specific educational tools or strategies that you feel would be most beneficial for you?
8	Are there any other areas within the ERAS program that you feel parents could be more involved in?
9	Do you have any other thoughts or comments that you would like to share?

*ERAS* enhanced recovery after surgery

The second phase (February 2020) consisted of follow-up with parents whose infants had been discharged. Phone calls were conducted by one author (JL). Conversations involved parental reflections on the discharge process, including areas necessitating improvement.

Notes and audio recordings were taken during all encounters and transcribed verbatim. An inductive or grounded theory approach was taken to qualitatively analyze transcripts using NVivo software (version 12) [21]. Two authors (MB, JL) coded concepts using a constant comparative approach to ensure consensus and reliability of codes. Concepts were grouped to identify themes emerging from the data.

## Results

**Fig. 2:**
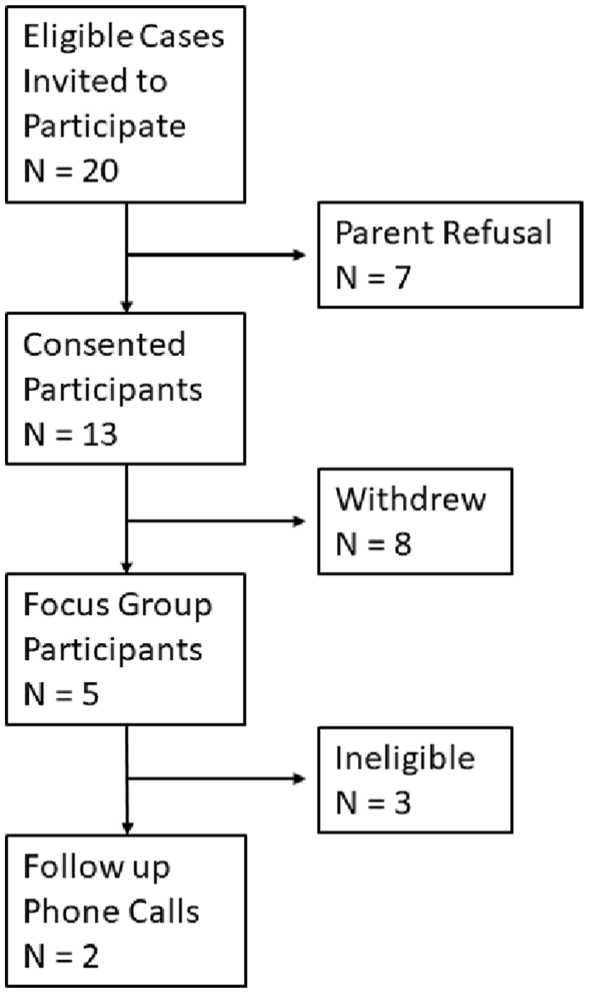
Overview of participant recruitment strategy

Of 20 eligible parents, 13 parents consented to participate. After confirming the focus group date, 8 parents withdrew, citing prior commitments, lack of child care, transportation issues, and leaving the city as withdrawal reasons. The focus group consisted of five parent participants: four mothers and one father (Fig. 2). Surgical diagnoses of the infants of the participants were congenital diaphragmatic hernia, intestinal atresias (including esophageal atresia and tracheoesophageal fistula), and gastroschisis.

All participants in the focus group had hospitalized infants. Follow-up phone calls were conducted 2 months later with two parents whose infants had subsequently been discharged. The children of the other three participants were still hospitalized (Fig. 2).

**Fig. 3:**
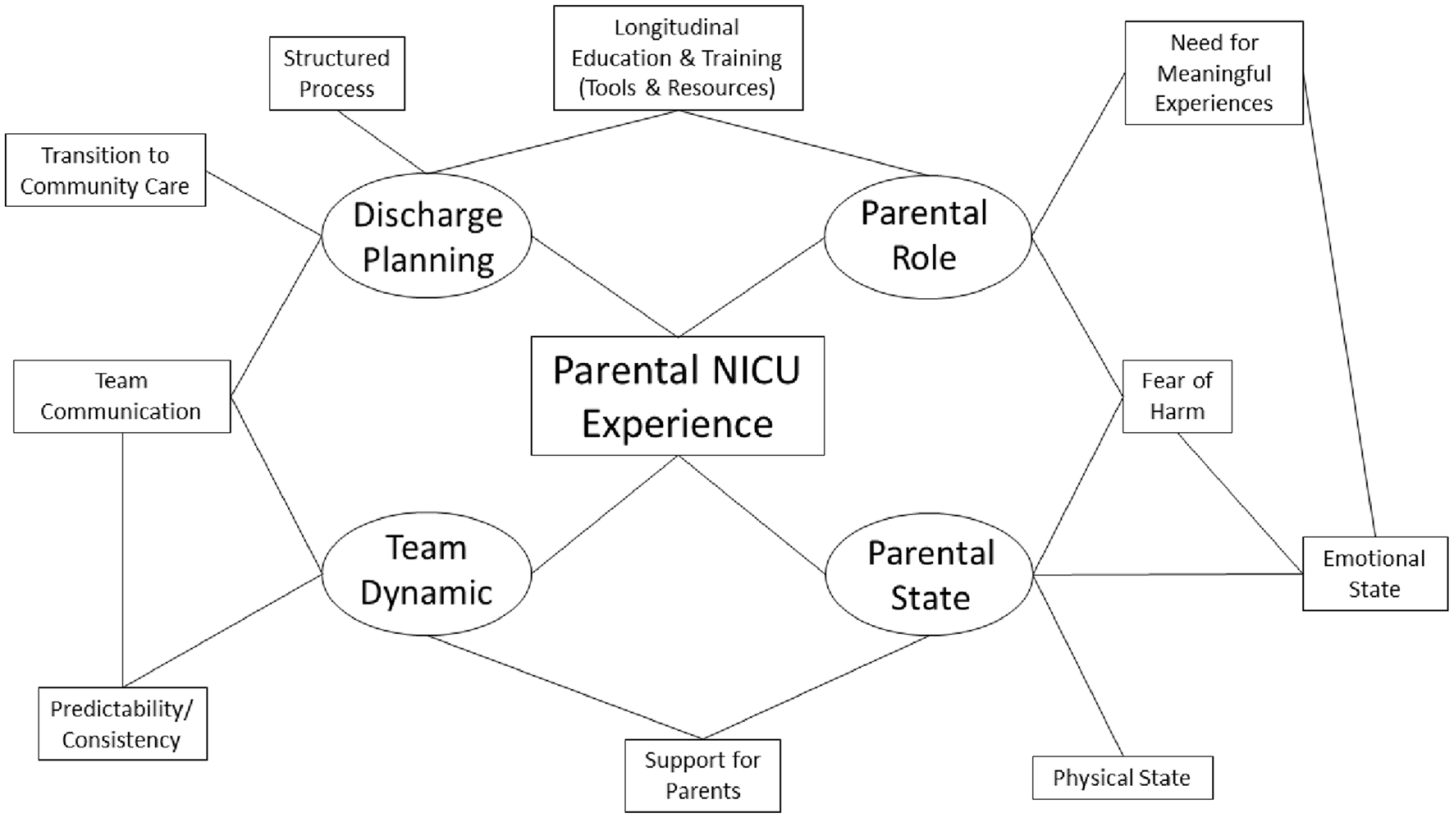
Themes and concepts identified by parents that contribute to the parental surgical NICU experience

Four major interrelated themes contributed to the parental surgical NICU experience: the parent’s personal state, parent’s perceived role as a caregiver, the care team dynamic, and the discharge preparation process (Fig. 3). Illustrative quotes are presented in Table 2.

**Table 2:** Illustrative quotes from parents of themes and concepts of the surgical NICU experience

Theme/concept	Illustrative quote
Parental physical state	“It took me two days to see my son that was in the same building because I wasn’t well enough …day five I was allowed to have a 4 h pass to come over [to see this one]” (1)
	“I’m groggy as anything. I was up for like 27 h” (3)
Parental emotional state	“With my first she wasn’t off me for ten full days. I didn’t leave my house. We stayed in bed. It was wonderful. To go from that to have barely touched her foot … It doesn’t feel real.” (2)
	“I’m terrified all the time that I’m going to do something to hurt her” (4)
Effective communication	“That whole day—she might have surgery, we’re not sure. It could be today, tomorrow, next week. It wasn’t until they were packing her up that I knew for sure. It was a little stressful” (2)
	“I don’t see how that could have got any better. He’s phenomenal. He’s really good with talking to people. It’s one thing to be an awesome surgeon but it’s another thing to have phenomenal people skills” (5)
	“In the beginning, you’re so emotional, you’ve got so much up here you can’t keep it all straight… to get a whole bunch of papers, I’m not reading that. You’re so fried all the time. You’re not sleeping. All this stuff going on. Now, I have to sit down and read all this and retain it to boot? Make them [more] fun, like a kid book. It’s easier to look at and listen to. The presentation can make a big difference” (1)
Support systems	“There’s a website that you share the link to and they subscribe for notifications … You can download an app to your phone, open it and I’m writing a journal entry.” (2)
	“It’s good for me… to get to know other moms who are kind of in a similar situation. Very rarely do you actually see any moms on the floor because usually [they’re] with their babies” (3)
Perceived pain	“In the back of my head I’m thinking, is this going to turn into an addiction later on?” (3)
Hands-on training	“I find the more you come, the more comfortable you are. A month ago, I had changed a diaper or 2. Now, I’m changing lots of diapers, I’m looking at the drains, measurements and weighing diapers, putting Aquaphor on her dry skin. I am more hands-on with the small things that I can do, because there’s still a lot that I can’t. I help with the assessment to try to keep her happy while they do their thing. With time, I feel more comfortable.” (4)
Caregiver role	“We’re so new. This is day six but I don’t know to ask for certain things, I’ve seen them do things and I wonder if I could do that but I don’t know where the lines are and I don’t want to ask for something that is [unreasonable].” (2)
Meaningful experiences	“I was able to hold him while they took the catheter out… make sure he was not too stressed out when they were doing that. It was kind of nice to be able to just be near him, touch him, do something, feel helpful. To be a mom. That’s pretty much it.” (3)
Care team dynamic	“At the end of rounds every morning they ask, ‘mom, dad, do you have any questions?’ That is your chance. That’s your moment and you get it every day… it’s really nice and I like how the docs do two weeks on. You get that consistency and continuity of care.” (4)
	“That was a little nerve-racking to not know who was going to be here when I couldn’t be.” (2)
Discharge planning	“Having us doing more skills in hospital before we’re discharged… Have us more hands-on and the docs, nurses watching us and then saying, yeah, you guys are ready. Making sure we’re ready before baby’s discharged because it’s a whole package.” (4)
	“We did feel quite prepared. And the reason why we can say that so confidently is because less than a week before, his twin brother was discharged. We had already gone through all of the discharge processes and procedures, so it was a bit of a unique situation. By the time [he was] coming home, we were very comfortable with already bringing one baby home. The only difference for him with the abdominal surgery was that the doctor had just said what to look for, what’s normal, what's not.” (1)

*NICU* neonatal intensive care unit, *NG* nasogastric, number in parentheses corresponds to focus group participant

### Parental state

Comments were grouped to identify factors that exacerbated a negative parental state or supported a positive parental state. Both the parent’s physical and emotional state impacted the surgical NICU experience. Parents reported being sleep deprived and hungry, while mothers described physical pain and health concerns related to labor and delivery that subsequently limited their interactions with their babies.

Participants experienced a variety of emotions including fear, guilt, anxiety, feeling disconnected from normal parental experiences, and a sense of accountability to update support systems. Often, these emotions stemmed from comparing experiences with previous children or ideas of what “normal” parenting could have been.

“You just feel guilty for not being there or doing enough … even [the fact] that they had been born early and all the issues they have” (3)

Effective communication with healthcare providers that included setting reasonable expectations and providing time to ask questions (e.g., prenatal counseling) comforted parents with the knowledge of what to expect and supported them in their role as part of the care team. When communication is lacking, uncertainty negatively impacted a parent’s emotional state, contributing to their fear, anxiety, and stress.

“We had a lot of time to ask questions … We knew exactly what was going to happen with the surgery. They had everything ready. There was a lot of planning that happened ahead of time so we felt really comfortable with that.” (1)

Effective verbal communication strategies to ensure knowledge transmission was extremely valued, especially in emergency and bad news scenarios. Conversely, the abundance of paperwork, overloaded with medical jargon, was perceived to be excessive early on.

“Just explaining everything and as terrifying as that conversation is, I walked away learning something. I knew what was happening. When you’re that emotional and you have that information in your face do you hear anything? I did. I walked away knowing it’s really bad right now but we might be able to turn this around and I held onto that.” (4)

Family support systems (relatives and friends) and formal activities promoting a parent community within the NICU generally had a positive impact on the parental state. However, parents felt burdened by the need to continuously update support systems.

A parent’s perception of their child’s pain from the surgical incision significantly affected their emotional state. All parents reported perceived pain and long-term effects of narcotic administration as major concerns and expressed the importance of medical education on perioperative opioid use.

“I always want to hold her but with a belly incision, I was scared to hurt her. There’s that fear that she can’t tell me she’s in pain” (2)

Parents desired involvement in caregiver activities; however, the perception of practicing on their child provoked anxiety. All parents felt that tools and strategies, particularly, simulations for practical skills would have been extremely beneficial.

“It was kind of scary to learn that I was practicing on my own child... It was really stressful and probably not the best way to put in your first NG tube. A simulation would be wonderful.” (2)

### Parental role

Table 3 illustrates the major categories elicited within this theme: the lack of normal parental experiences and the need for alternative meaningful parental experiences despite the abnormal environment.

**Table 3:** Factors that contribute to a parent’s perception of their role in the NICU

Lack of normal experience		Meaningful experiences	
Emotions	Barriers	Opportunities	Facilitators
Guilt	Incubator	Holding/skin-to-skin	Effective communication
Stress	Lines/tubes	Family pictures	Staff support
Disconnected/not like a parent	Infant pain/perceived harm	Celebrate milestones/ holidays	Hands-on and graduated training
Fear/terror	Uncertainty of role	Balloons/decorations/gifts	Use of various tools/strategies
Anxiety	Perception of needing medical team approval	Involvement with assessments	
		Participate in caregiver activities	

*NICU* neonatal intensive care unit

The main barriers to a normal parent–child connection were physical in nature. Psychological barriers also existed including the uncertainty of the parent role and the belief that authorization was required to provide even routine care. The perception of pain and fear that they may do something wrong significantly contributed to a parent’s inability to take on a normal role. As a result of the altered parental experience, families reported many emotions, the most common was feeling disconnected or not like a parent (Table 3).

“It takes forever to get there and this is all I can do, stand here and stare at her. To hold her, we need RT, another nurse. Someone’s got to hold her line, move the vent tube, get the chair ready. It’s a five-man thing just to hold your kid for an hour …2 months later, I still feel like I’m not her mother because I don’t live with her. I don’t just pick her up… we don’t even have a picture of the three of us. I feel like I got ripped off.” (4)

Despite the limitations of the NICU environment, parents identified meaningful opportunities that positively impacted the parent–child bond. These included things as simple as family photos and celebrating milestones and holidays, while others involved creating roles for parents during assessments and caregiver activities. Staff support and directed hands-on training provided parents with confidence in their caregiver role.

“For Thanksgiving, they gave me this little card that she “made” overnight. It was her footprints. It was huge. When I saw those footprints and the nurse [said,] ‘those are really her footprints.’ That was it, waterworks. I bawled for three hours. It was wonderful.” (4)

### Care team dynamics

Within the care team, parents stated that consistency of care and timely communication with effective knowledge transmission allowed parents to build rapport with the medical team leading to trust and a sense of partnership. Poor communication and uncertainty were detrimental to that relationship. Early communication, even if plans were incomplete, was favored over the lack of information.

“All of a sudden our baby was being moved in ten minutes and maybe having surgery … I felt like they probably could have told us a little bit more in the days upcoming.” (4)

Parents appreciated the opportunity to take an active role in decision-making during daily rounds. The uncertainty that parents often felt about their role improved when staff members consistently offered opportunities to be involved in assessments and care. A NICU orientation, including the various monitors and lines, supported familiarity to the new setting. Parents valued consistency in care and were uncomfortable when they were unfamiliar with the team looking after their child.

### Planning and preparation for discharge

Discharge preparation occurs throughout the hospital stay from admission until care was transitioned to the community setting. Parents valued communication, education, and graduated, hands-on training throughout the hospital stay, particularly, the opportunity to perform skills under direct supervision of an expert.

Although much of the preparation for discharge is done on preceding days, it is still extremely important to continue with effective and timely communication on the day of discharge to ensure a smooth and seamless process. If poorly executed, the day of discharge can leave a negative memory for parents, breaching the trust and relationship that was previously developed.

“That was really frustrating because we were scared to leave, to go eat or get a coffee, we didn't want to miss rounds. We were waiting for hours… To be forgotten about with rounds the day you're being discharged is kind of disappointing… it was not the way we wanted to say goodbye.” (2)

One mother suggested that a structured process for the day of discharge could ease anxiety by providing expectations for families. This could include an expected timeline and checklist of people to see and items to complete.

Transitioning to the community setting provoked significant anxiety in parents. Home visits by a nurse and home monitors were considered as anxiety relievers. Confidence in the education and training received throughout their stay comforted parents at discharge. Aspects that further eased the transition were knowledge of contact information, clinic follow-ups, and transfer of medical information to their pediatricians.

## Discussion

Our study provides the first qualitative report of the parents’ perspective of the surgery NICU experience. Four major themes included the parental state, altered parent caregiver role, care team dynamic, and discharge planning process.

In the early postpartum period, parents struggle to cope with the stresses of their newborn undergoing surgery [1, 2, 4, 7, 8, 11]. Much of the previously published literature has been survey based using Likert scale questionnaires which inherently restrict participant responses or relate to preterm infants where the major barriers to developing the parent–infant bond are physical issues surrounding the incubator, lines, and tubes [1–8, 10, 11, 13]. Our parent participants described similar difficulties associated with physical barriers inherent in the NICU environment but also report additional significant stressors related to perioperative pain [2, 4, 8, 11]. Parents report stresses related to their inability to protect their child from pain as well as fears that their involvement as inexperienced caregivers could cause further pain above that already endured from surgery [11]. Participants discussed their initial lack of knowledge around narcotics and the concerns they had with potential for long-term consequences such as addiction when their infants were provided with narcotic analgesics postoperatively. Our participants reported that staff support and education helped parents build confidence to participate as caregivers and understand their child’s needs with regard to perioperative pain management. These findings were incorporated into the neonatal ERAS® implementation strategy where particular attention was paid toward effective communication, education, and support to parents, especially with regard to perioperative pain and narcotic requirements.

Parents reported that developing relationships with other NICU parents and belonging to the NICU community provided a positive impact on their personal states. Interestingly, we had the unique ability to compare different environments and the impact the environment had on families. Patients in this study were transferred from a traditional large open-concept NICU to a surgical NICU made up entirely of individual rooms. Parents enjoyed the privacy of individual rooms, but occasionally felt isolated. The large, primarily medical NICU holds weekly education sessions for parents and families covering medical and prematurity topics, further allowing for interactions between NICU families. Participants felt that regularly scheduled educational sessions similar to those taking place at the large, primarily medical NICU would be beneficial at the surgical NICU if they covered surgical rather than prematurity topics. The opportunity to convene and develop a parent community is especially important in a NICU with individual rooms.

Despite the altered parental role, parents described many opportunities for meaningful experiences. Families were deeply grateful for the effort that staff made to help celebrate milestones, facilitate family photos, and create memorabilia. Parents were intimidated by the procedures involved in even routine care, but found hands-on training and purposeful, graduated practice of skills helped to build confidence in their abilities [9, 22]. There was enthusiasm for different educational modalities, specifically, simulation to ease the anxiety of learning and practicing new skills.

Trust was built through family-centered daily rounds, where parents were encouraged to ask questions and participate in shared decision-making [7, 22]. Timely and effective communication was a major facilitator of optimal care team dynamics and was especially important in emergency settings and bad news scenarios. Early and thoughtful explanation and communication including reasonable expectations of next steps in care may ease parent anxiety [3, 8]. Conversely, parents described a sense of information overload from receiving an abundance of paperwork filled with medical jargon. Instead, they requested family-friendly documents that would be more easily digested. As a result, a family-friendly education book was created as part of the ERAS^®^ implementation strategy that incorporated information deemed important based on the parent participants, including, but not limited to, information surrounding the NICU environment and corresponding care providers, intestinal surgery basics including information on ostomies, perioperative pain control, their role as part of the care team, expected course of recovery, and discharge planning.

Providing education and training longitudinally throughout the hospital stay was equally important to that received on the day of discharge [9, 22]. When performed well, parents expressed satisfaction and comfort with the discharge process [3]. Parents suggested that a discharge checklist and timeline for the day of discharge may provide structure to the process [22].

Concepts spanning across themes included the need for effective communication, involvement of parents in caregiving responsibilities and shared decision-making as well as developing skills and confidence through longitudinal hands-on training. These are all core elements of the neonatal ERAS® guideline [16]. Participant suggestions from this focus group, including a family-friendly education booklet to introduce the surgical NICU, perioperative course, and pain management, as well as a strategy for parent education and engagement throughout the entire perioperative journey, were included into our institution’s neonatal ERAS® implementation strategy [20].

We were limited by the small number of participants in our focus group. Successful focus groups rely on open and equal participation of all parties present, necessitating a small group setting and English proficiency, but limiting generalizability of findings. Family commitments as well as distance needed to travel impeded parents from attending the focus group once discharged. We predominately had female participants and all were Caucasian and of traditional mother–father families, as such, results may not be generalizable to other ethnicities, cultures, and family structures.

We present a unique, qualitative parent perspective of the surgical NICU experience. Previous studies have mainly explored premature, non-operative infants who are inherently different than surgical neonates [6, 8, 10, 11, 13]. Other reports have targeted cardiac surgery at various ages and differing intervals from surgery or have primarily used surveys which restrict participant responses [1, 2, 4, 8, 11]. Our approach utilizing a focus group allowed themes to naturally develop through conversation among the participants, ensuring that perspectives, experiences, and what mattered most to parents were captured. Too often, clinicians tell patients and their families what we think is important for them, rather than listening to what they need [23]. A quote from one parent is a perfect example, “She didn't really seem to actually answer any of my questions. She would just talk about things that sounded like they were maybe related to what I wanted to know, but not actually answer anything.” To actualize family-centered care, we have to ask the right people the right questions and truly listen to the answers; otherwise, we may overlook what is significant to families.
